# Is strain by Speckle Tracking Echocardiography dependent on user controlled spatial and temporal smoothing? An experimental porcine study

**DOI:** 10.1186/1476-7120-11-32

**Published:** 2013-08-22

**Authors:** Christian Arvei Moen, Pirjo-Riitta Salminen, Geir Olav Dahle, Johannes Just Hjertaas, Ketil Grong, Knut Matre

**Affiliations:** 1 Department of Clinical Science, University of Bergen, Haukeland University Hospital, Bergen NO-5021, Norway; 2 Section of Cardiothoracic Surgery, Department of Heart Disease, Haukeland University Hospital, Bergen, Norway

**Keywords:** Myocardium, Deformation, Strain, Left ventricular function, Ischemia, Pigs, Experimental, Speckle tracking echocardiography, Spatial smoothing, Temporal smoothing

## Abstract

**Background:**

Speckle Tracking Echocardiography (STE) strain analysis relies on both spatial and temporal smoothing. The user is often allowed to adjust these smoothing parameters during analysis. This experimental study investigates how different degrees of user controllable spatial and temporal smoothing affect global and regional STE strain values in recordings obtained from normal and ischemic myocardium.

**Methods:**

In seven anesthetized pigs, left ventricular short- and long-axis B-mode cineloops were recorded before and after left anterior descending coronary artery occlusion. Peak- and postsystolic global STE strain in the radial, circumferential and longitudinal direction as well as corresponding regional strain in the anterior and posterior walls were measured. During post-processing, strain values were obtained with three different degrees of both spatial and temporal smoothing (minimum, factory default and maximum), resulting in nine different combinations.

**Results:**

All parameters for global and regional longitudinal strain were unaffected by adjustments of spatial and temporal smoothing in both normal and ischemic myocardium. Radial and circumferential strain depended on smoothing to a variable extent, radial strain being most affected. However, in both directions the different combinations of smoothing did only result in relatively small changes in the strain values. Overall, the maximal strain difference was found in normal myocardium for peak systolic radial strain of the posterior wall where strain was 22.0 ± 2.2% with minimal spatial and maximal temporal smoothing and 30.9 ± 2.6% with maximal spatial and minimal temporal smoothing (P < 0.05).

**Conclusions:**

Longitudinal strain was unaffected by different degrees of user controlled smoothing. Radial and circumferential strain depended on the degree of smoothing. However, in most cases these changes were small and would not lead to altered conclusions in a clinical setting. Furthermore, smoothing did not affect strain variance. For all strain parameters, variance remained within the corresponding interobserver variance.

## Background

Speckle Tracking Echocardiography (STE) has become an important research tool for studying myocardial function [1-5]. The STE strain algorithm relies heavily on both spatial and temporal smoothing, methods for fitting a smooth curve to a set of noisy observations [1,6-9]. Without smoothing, a strain curve displayed by the software would go through all the measured data points. However, due to the noise, such a curve could contain many fluctuations that would belie the nature of the underlying true strain curve. Increasing the smoothing would make the strain curve depart from the measured data points, but would result in a smoother curve. The user is often allowed to adjust the degree of these smoothing parameters when analyzing the recordings [1]. Altering these settings may affect the calculated segmental strain values [4]. To our knowledge, no study has investigated to what extent adjustment of user controlled spatial and temporal smoothing will affect global as well as regional STE strain values in recordings from normal and ischemic myocardium.

This experimental study was undertaken to investigate how different smoothing settings affected global and regional STE strain values for one commonly used STE algorithm. Echocardiographic recordings were obtained in normal myocardium and also after left anterior descending (LAD) coronary artery occlusion. STE analysis was performed to obtain left ventricular (LV) global radial, circumferential and longitudinal strain as well as corresponding regional strain in the LV anterior and posterior wall. During post-processing, strain analysis was performed with three different degrees of both spatial and temporal smoothing (minimum, factory default and maximum), resulting in a total of nine different combinations. For each echocardiographic view, the STE algorithm divides the myocardium into six segments and automatically estimates average strain within each segment before the strain curve is displayed. A preset amount of smoothing is therefore built into the algorithm and not controllable by the user [6,7]. We thus hypothesized that the amount of user controlled spatial and temporal smoothing applied during analysis would have limited effects on the resulting strain estimates.

An experimental open chest model was chosen to repeatedly obtain recordings of similar image quality. The model also allowed well defined and standardized ischemic regions, resulting in minimal variation in strain values between hearts for both the normal and the ischemic situation. The current model therefore enabled minimization of the variation in factors that potentially could have overshadowed the effect of altering smoothing on the strain values.

## Methods

### Experimental preparation

This study included seven young pigs (Norwegian Land Race) of either sex, weighing 40 ± 2 (SD) kg and a calculated body surface area of 1.06 ± 0.04 (SD) m^2^. The protocol was approved by the Norwegian State Commission for Laboratory Animals (project No. 20092088). Procedures were performed in accordance with the European Communities Council Directive 2010/63/EU.

After premedication with a mixture of ketamine, diazepam and atropine, anesthesia was induced and maintained by loading doses and continuous infusions of fentanyl, sodium pentobarbital, midazolam and pancuronium as previously described in detail [10,11]. The animals then underwent tracheotomy and intubation. Mechanical ventilation (Cato M32000, Drägerwerk, Lübeck, Germany) was sustained with a combination of N_2_O (56-57%) and oxygen. Heart rate was monitored by a surface ECG. The abdominal aorta and inferior caval vein were cannulated via the right femoral artery and vein for blood sampling and infusion purposes. Temperature was measured and urine drained with a catheter–thermistor inserted into the bladder. Midline sterno- and pericardiotomy were performed, and a pressure-tip catheter (MPC-500, Millar, Houston, TX) was inserted into the LV through the apex. Peak systolic and end-diastolic pressures as well as maximal (dP/dt_max_) and minimal (dP/dt_min_) first derivative of LV pressure were measured. An identical catheter was placed in the aorta through the internal mammary artery for measurement of central aortic pressure. Continuous cardiac output was measured with a 7.5 F balloon floating catheter (Swan-Ganz CCO/VIP, Edward Lifesciences, Irvine, CA) placed in the pulmonary artery and connected to a cardiac output computer (Vigilance, Edward Lifesciences). The LAD coronary artery was then dissected free from underlying tissue between the first and second diagonal branch to allow external clamping of the artery. Animals were allowed a 20 min stabilization period after instrumentation was completed.

### Protocol

A baseline registration was followed by a new set of registrations 10 min after LAD coronary artery occlusion. For each situation, arterial blood gases, global hemodynamics and echocardiographic recordings were obtained. The animals were then used for another protocol, a pilot study of cardiac function after reperfusion following LAD coronary artery occlusion.

### Echocardiography

**Figure 1:**
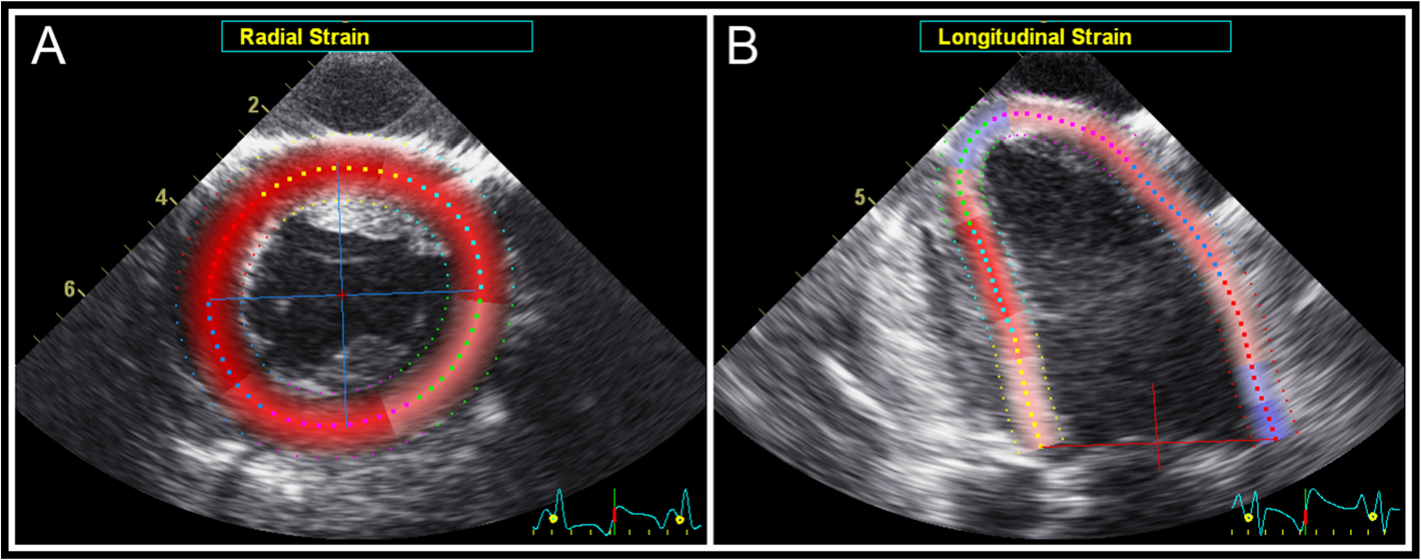
**Speckle Tracking Echocardiography (STE). (A)** Epicardial left ventricular short-axis view used for STE. During analysis, knots (square-like points) are evenly distributed along the middle of the region of interest and the region of interest is itself divided into six standardized segments. Tracking of these knots from frame to frame is used for estimation of radial and circumferential strain in each segment. The anterior and posterior wall was defined as the yellow and pink segment, respectively. **(B)** Epicardial left ventricular long-axis view used for STE. The same principle is used for estimation of longitudinal strain in each segment. The anterior and posterior wall was defined as the dark blue and cyan segment, respectively.

A soft silicone pad (3×3×1.5 cm), placed between the probe and the epicardium acted as a cushion for the moving heart and as an offset to reduce near field artifacts [12]. Cineloops were recorded on a digital ultrasound scanner (Vivid 7 Pro, GE Vingmed Ultrasound, Horten, Norway) using a 10 MHz phased array transducer (10S, GE Vingmed Ultrasound). This enabled a frequency of 8–10 MHz for the B-mode recordings. By narrowing the image sector and reducing depth, a B-mode short-axis view (81–107 frames/s) half way between the ventricular equator and apex was obtained for later STE strain analysis in the radial and circumferential direction (Figure 1A). This view included both the anterior wall (affected by the occlusion) and the posterior wall (not affected by the occlusion). An apical long-axis view (71–91 frames/s), visualizing the part of the anterior wall affected by the occlusion in long-axis, was then recorded for later STE strain analysis in the longitudinal direction (Figure 1B). Pulsed wave Doppler (PWD) recordings in the aortic orifice were used to identify the timing of aortic valve opening and closure. All recordings were done during brief stops (5–6 beats) of the respirator at end-expirium. No changes in hemodynamics could be seen during these short respirator stops.

### Data analysis

All recordings were analyzed using EchoPac PC BT11 software (GE Vingmed Ultrasound). For STE analysis of the short- and long-axis view, manual tracing of the endocardium and individual adjustment the ROI width to only include the myocardium for each recording was performed. During analysis, knots are automatically and evenly distributed around the middle of the ROI while the ROI itself is divided into six standard segments (Figure 1, knots are the square-like points in the middle of the ROI). The software calculates local velocity of each knot, in each frame throughout the heart cycle, by weighted linear interpolation of multiple velocities detected by block matching between adjacent frames in the vicinity of each knot [1,8,13,14]. This stage is not user controllable. Thereafter, to obtain a smooth curve for movement of each knot throughout the heart cycle, as well as a smooth transition between adjacent knots, cubic spline smoothing is performed in both space and time. This smoothing procedure is explained and illustrated in more detail elsewhere [15].

**Figure 2:**
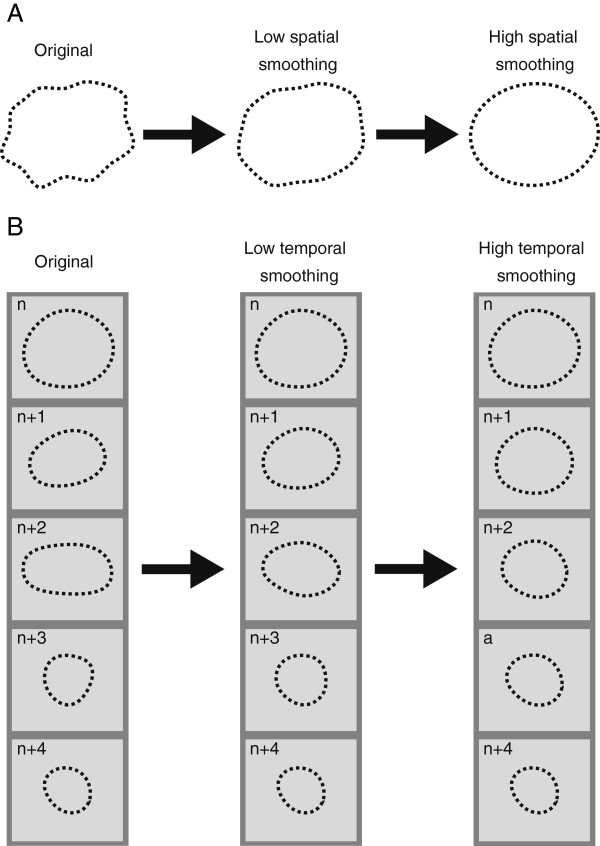
**Illustration of (A) spatial and (B) temporal smoothing of a contour created by the points in the middle of the region of interest (ROI), as shown in Figure**1**A.** To obtain a regularly moving ROI throughout the heart cycle, the smoothing procedure restricts the ROI from large or irregular movements from frame to frame. In **(A)**, increasing the spatial smoothing will, for each separate frame of the heart cycle, reduce kinks or steps in the contour. With smooth contours at each frame, increasing the temporal smoothing will, on the other hand, enable a smooth transition between contours from frame to frame, as indicated in the rightmost column of **(B)**. Arrows indicate increasing degree of smoothing.

The spatial smoothing is performed by applying cubic spline smoothing to the entire chain of knots in each frame separately (Figure 2A). Spatial smoothing also use tracking weights, meaning that the level of smoothing is reduced in areas of reliable tracking and increased in areas of bad tracking [16]. With minimum spatial smoothing, the spatial resolution of the estimates is approximately equal to the dimension of the ROI. Maximum spatial smoothing, however, is determined by an experimental constant not provided by the manufacturer.

The temporal smoothing, on the other hand, is performed by applying cubic spline smoothing to the entire chain of frames for each knot separately (Figure 2B). Minimum temporal smoothing is approximately equal to the time from one frame to the next. Again, maximum temporal smoothing is determined by an experimental constant not provided by the manufacturer.

Due to the inherent reduced tracking quality in short-axis views, spatial and temporal smoothing levels in the short-axis views exceed those in the long-axis views (before image processing, the software must be told what kind of image projection is being analyzed).

**Figure 3:**
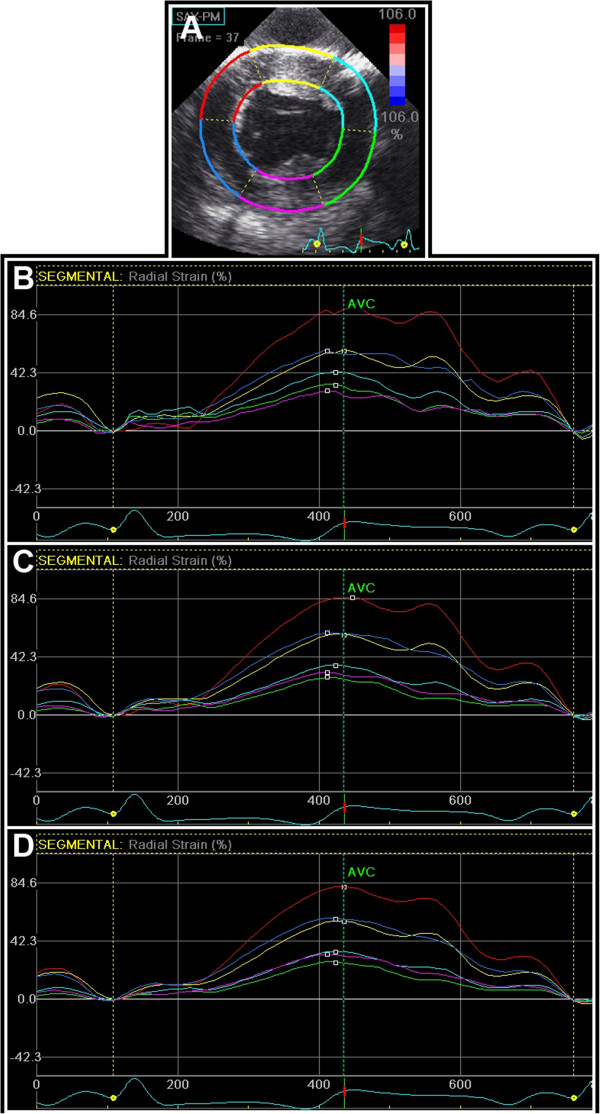
**Radial strain curves with different degrees of user controlled spatial and temporal smoothing. (A)** Epicardial left ventricular short-axis view used for 2-dimensional strain analysis with Speckle Tracking Echocardiography. The left ventricle is divided into 6 standard segments, labeled AntSept (yellow), Ant (cyan), Lat (green), Post (pink), Inf (blue) and Sept (red). Regional radial and circumferential strain was measured within each segment. The anterior and posterior wall was defined as the AntSept and Post segment, respectively. Global strain was calculated as the average value of the six segmental strain values at each frame throughout the heart cycle. The curves show radial strain at baseline with **(B)** minimal, **(C)** factory default and **(D)** maximal degrees of both spatial and temporal smoothing.

In trace mode, start of integration was moved to start of the QRS-complex and end-systole was defined and imported from the PWD recording. All recordings were analyzed with the drift compensation turned on. In results mode, average strain curves within each of the six preset regions of the myocardium were then generated by the software (Figure 3). Peak systolic, end-systolic and postsystolic radial, circumferential and longitudinal strain in the six segments were reported for nine different combinations of spatial and temporal smoothing. These settings were created by in turn adjusting both spatial and temporal smoothing to minimum, factory default and maximum. Postsystolic strain was derived from the difference between end-systolic strain and the postsystolic peak of the strain curve. If no postsystolic peak was present, postsystolic strain was set to zero [17,18].

For each of the nine smoothing settings, peak- and postsystolic global strain was obtained from calculation of the average strain from the six strain curves at each frame throughout the heart cycle.

The short-axis B-mode view was also used for measurement of wall thickening in the anterior and posterior wall. Diastolic wall thickness (WT_dia_) was measured at the start of the QRS complex, while systolic wall thickness (WT_sys_) was measured at aortic valve closure.

### Statistical analysis

Unless otherwise noted, all data are expressed as mean ± SEM. Statistical analysis was performed using commercial software (PASW Statistics 18, SPSS Inc, Chicago, IL). Hemodynamics and wall thickening were analyzed by paired sample t-tests. STE strain variables with different settings for both spatial and temporal smoothing were analyzed by two-way ANOVA for related measurements. This enabled extraction of separate P-values for spatial (P_s_) and temporal (P_t_) smoothing as well as for the interaction between these two parameters (P_i_). If the Mauchly’s test of sphericity was significant (P < 0.05), the Greenhouse-Geisser adjustment of degrees of freedom was selected. Cell means were compared with Newman-Keuls multiple contrast tests when appropriate.

The interobserver variability for STE strain was estimated by comparing all primary results using default software settings with corresponding de novo measurements by one of the authors who had not participated during the primary analysis. To further examine how different smoothing settings affected the interobserver agreement, measurements were also compared for minimum as well as for maximum spatial and temporal smoothing. Estimates are given as mean difference ± SD as well as the intraclass correlation coefficient (R_ic_).

A comparison between strain variance and corresponding interobserver variance was then performed. First, for each strain parameter, the nine different settings were tested for equal variance using Levene’s test of homogeneity of variances. If equal variance could be assumed, the overall variance of the nine settings was tested against the interobserver variance (for default software settings) using a test for the difference between two variances [19]. For all analyses a P-value < 0.05 was considered statistically significant.

## Results

### Blood gases and hemodynamics

Arterial blood gas analysis throughout the protocol showed stable respiratory conditions with normal values for this pig model [11]. This was also the case for end-tidal levels of oxygen, carbon-dioxide and nitrous oxide and for hemoglobin, rectal temperature and diuresis.

Hemodynamics are shown in Table 1. Compared to preocclusion values, LVEDP increased and LVSP_max_, dP/dt_max_, cardiac index and mean aortic pressure decreased while dP/dt_min_ was less negative following LAD occlusion. Heart rate did not change (P = 0.65).

**Table 1:** Hemodynamic values for seven pigs before and after left anterior descending coronary artery occlusion

	**Baseline**	**Occlusion**	**Statistics**
Heart rate, beats/min	92 ± 5	90 ± 3	P = 0.65
LVSP_max_, mmHg	121 ± 2	96 ± 5*	P < 0.001
LVEDP, mmHg	7.7 ± 0.7	14.1 ± 1.4*	P = 0.003
dP/dt_max_, mmHg/s	1446 ± 57	1241 ± 109*	P = 0.027
dP/dt_min_, mmHg/s	-1941 ± 73	-1390 ± 92*	P < 0.001
Cardiac index, L/min per m^2^	4.2 ± 0.2	3.6 ± 0.3*	P = 0.023
MAP, mmHg	100 ± 4	76 ± 6*	P < 0.001

Mean ± SEM. n = 7. LVSP_max_ and LVEDP = left ventricular peak systolic and –end-diastolic pressure; dP/dt_max_ and dP/dt_min_ = peak positive and peak negative first derivatives of left ventricular pressure; MAP = mean aortic pressure; *denotes significant difference from baseline value by paired sample t-test.

### Wall thickening

In the anterior wall, WT_dia_ was 6.9 ± 0.3 mm at baseline and decreased to 5.9 ± 0.3 during occlusion (P = 0.04). WT_sys_ was 9.9 ± 0.3 mm at baseline and decreased to 5.7 ± 0.2 mm during occlusion (P < 0.001). Anterior wall thickening was 44 ± 2% at baseline and decreased to −2 ± 2% during occlusion (P < 0.001). In the posterior wall, WT_dia_ was 7.9 ± 0.1 mm and WT_sys_ was 10.2 ± 0.3 mm at baseline. Both remained unchanged (P = 0.20 and 0.23). Posterior wall thickening was 31 ± 2% at baseline and also remained unchanged (P = 0.99).

### Global STE strain

**Figure 4:**
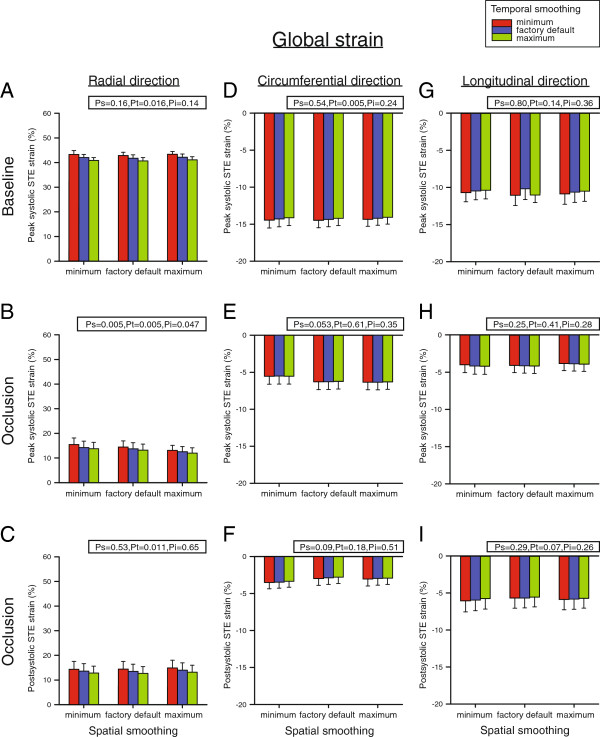
**Peak- and postsystolic global strain by Speckle Tracking Echocardiography (STE) in the radial (A - C), circumferential (D - F) and longitudinal (G - I) direction at baseline and after left anterior descending coronary artery occlusion.** Measurements were performed with nine different combinations of spatial and temporal smoothing created by setting these parameters in turn to minimum, factory default and maximum. Mean values of seven animals, error bars represent SEM. P_s_, P_t_ and P_i_ = P-value for the effect of spatial smoothing, temporal smoothing and interaction between these two adjustments, respectively.

In the radial direction, peak systolic strain at baseline decreased with increasing temporal smoothing (P_t_ = 0.016) (Figure 4A). Strain values with minimum smoothing were higher than values with both default and maximum smoothing and default values were again higher than values with maximum smoothing (P < 0.05). No postsystolic strain was found at baseline. After occlusion, the effect of temporal smoothing depended on the level of spatial smoothing (P_i_ = 0.047) (Figure 4B). The maximal calculated strain value (15.5 ± 2.6%) was obtained with both minimal spatial and temporal smoothing, whereas the minimal value (12.0 ± 2.1%) was measured with both maximal spatial and temporal smoothing. Postsystolic radial strain also depended on the amount of temporal smoothing (P_t_ = 0.011) (Figure 4C). These strain values were higher with minimal smoothing than with both default and maximum smoothing and default strain values were again higher than values with maximum smoothing (P < 0.05).

In the circumferential direction, peak systolic strain at baseline also decreased (less negative) with increasing temporal smoothing (P_t_ = 0.005) (Figure 4D). Strain values calculated with minimal smoothing were higher (more negative) than values with both default and maximum smoothing and default strain values were again higher than with maximum smoothing (P < 0.05). No postsystolic strain was found at baseline. During occlusion, both peak systolic and postsystolic strain values remained unaffected by smoothing and averaged −6.0 ± 1.0% and −3.1 ± 0.9%, respectively (Figure 4E and F).

In the longitudinal direction, peak systolic strain at baseline was not altered by adjusting either temporal or spatial smoothing and averaged −10.6 ± 1.3% (Figure 4G). Postsystolic strain was absent at baseline. During occlusion, both peak systolic and postsystolic strain values also remained unaffected by smoothing and averaged −4.0 ± 1.0% and −5.8 ± 1.4%, respectively (Figure 4H and I).

### Regional radial STE strain

**Figure 5:**
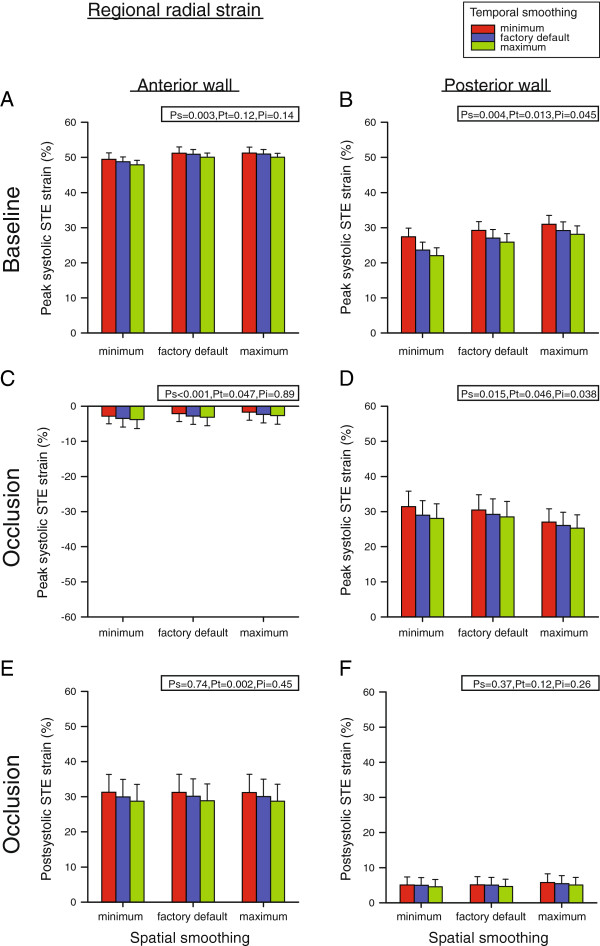
**Regional radial peak- and postsystolic Speckle Tracking Echocardiography (STE) strain in the anterior and posterior myocardial wall at baseline (A and B) and after left anterior descending coronary artery occlusion (C - F).** Measurements were performed with nine different combinations of spatial and temporal smoothing created by setting these parameters in turn to minimum, factory default and maximum. Mean values of seven animals, error bars represent SEM. P_s_, P_t_ and P_i_ as defined in Figure 4.

Radial peak systolic STE strain in the anterior wall at baseline increased with increasing spatial smoothing (P_s_ = 0.003) (Figure 5A). Values with minimum spatial smoothing were lower than values found with both default and maximum smoothing (P < 0.05). In the posterior wall, however, temporal smoothing affected strain depending on the level of spatial smoothing (P_i_ = 0.045) (Figure 5B). The minimal calculated strain value (22.0 ± 2.2%) was obtained with minimal spatial and maximal temporal smoothing, whereas the maximum value (30.9 ± 2.6%) was measured with maximal spatial and minimal temporal smoothing.

During occlusion, anterior wall peak systolic strain depended on both temporal and spatial smoothing independently (P_s_ < 0.001 and P_t_ = 0.047) (Figure 5C). For spatial smoothing, strain values with minimal smoothing were more negative than with both default and maximum smoothing, and default values were again more negative than with maximum smoothing (P < 0.05). For temporal smoothing, strain values with minimum smoothing were less negative than default values which were again less negative compared to values with maximum smoothing (P < 0.05). In the posterior wall, temporal smoothing affected strain depending on the level of spatial smoothing also following LAD occlusion (P_i_ = 0.038) (Figure 5D). The minimal calculated strain value (25.3 ± 3.8%) was obtained with both maximal spatial and temporal smoothing, whereas the maximum value (31.4 ± 4.2%) was measured with both minimal spatial and temporal smoothing.

Postsystolic strain was not present in any segment at baseline. During occlusion, postsystolic strain in the anterior wall depended on the degree of temporal smoothing with maximal strain estimates obtained with minimal temporal smoothing (P_t_ = 0.002) (Figure 5E). Minimum temporal smoothing resulted in higher strain values than with both default and maximum smoothing, and values were also higher with default compared to maximum temporal smoothing (P < 0.05). Postsystolic strain in the posterior wall was low, did not depend on smoothing and averaged 5.1 ± 2.2% (Figure 5F).

### Regional circumferential STE strain

**Figure 6:**
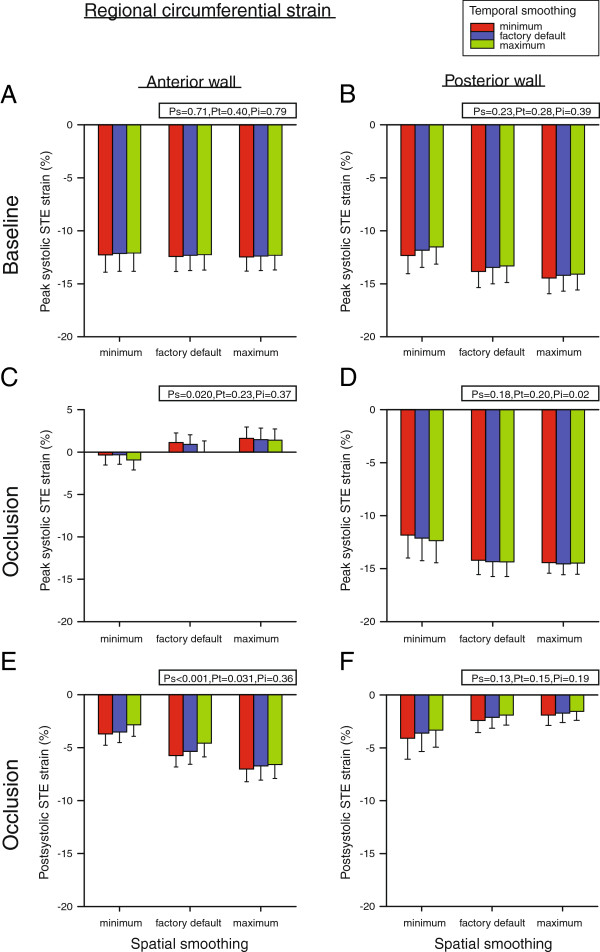
**Regional circumferential peak- and postsystolic Speckle Tracking Echocardiography (STE) strain in the anterior and posterior myocardial wall at baseline (A and B) and after left anterior descending coronary artery occlusion (C - F).** Measurements were performed with nine different combinations of spatial and temporal smoothing created by setting these parameters in turn to minimum, factory default and maximum. Mean values of seven animals, error bars represent SEM. P_s_, P_t_ and P_i_ as defined in Figure 4.

Circumferential peak systolic STE strain in the anterior wall at baseline was not altered, neither by temporal nor by spatial smoothing, and averaged −12.3 ± 1.5% (Figure 6A). This was also the case for the posterior wall, where strain averaged −13.4 ± 1.4% (Figure 6B). During occlusion, peak systolic strain in the anterior wall depended on spatial smoothing alone (P_s_ = 0.020) (Figure 6C). Values calculated with minimum smoothing were negative and different from the positive values for both default and maximum smoothing (P < 0.05). Strain values with default smoothing were also lower than with maximum smoothing (P < 0.05). In the posterior wall, temporal smoothing affected strain depending on the degree of spatial smoothing (P_i_ = 0.02) (Figure 6D).

Postsystolic strain was absent in both the anterior and posterior wall at baseline. During occlusion, postsystolic strain in the anterior wall depended on both spatial and temporal smoothing independently (P_s_ < 0.001 and P_t_ = 0.031) (Figure 6E). For spatial smoothing, minimum smoothing resulted in less negative postsystolic strain values than with both default and maximum smoothing, and values with default were less negative than values with maximum smoothing (P < 0.05). For all levels of spatial smoothing, increasing temporal smoothing reduced strain (P < 0.05). Postsystolic strain in the posterior wall remained unaffected by smoothing and averaged −2.5 ± 1.2% (Figure 6F).

### Regional longitudinal STE strain

**Figure 7:**
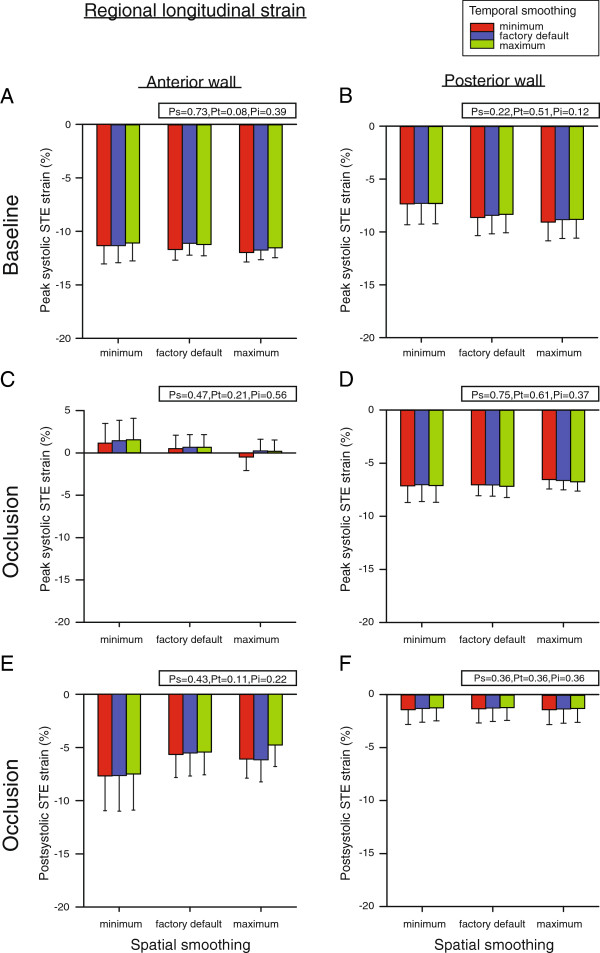
**Regional longitudinal peak- and postsystolic Speckle Tracking Echocardiography (STE) strain in the anterior and posterior myocardial wall at baseline (A and B) and after left anterior descending coronary artery occlusion (C - F).** Measurements were performed with nine different combinations of spatial and temporal smoothing created by setting these parameters in turn to minimum, factory default and maximum. Mean values of seven animals, error bars represent SEM. P_s_, P_t_ and P_i_ as defined in Figure 4.

Longitudinal peak systolic STE strain in the anterior wall at baseline was not altered by adjusting either temporal or spatial smoothing and averaged −11.4 ± 1.1% (Figure 7A). This was also the case for the posterior wall where peak systolic strain averaged −8.2 ± 1.8% (Figure 7B). During occlusion, values also remained unaffected by smoothing. In the anterior wall, strain averaged 0.7 ± 1.7% (Figure 7C). In the posterior wall, strain averaged −6.9 ± 1.0% (Figure 7D). Postsystolic strain was absent in both segments at baseline. During occlusion, postsystolic strain in the anterior and posterior wall did not dependent on smoothing and averaged −6.3 ± 2.3% (Figure 7E) and −1.3 ± 1.3% (Figure 7F), respectively.

### Interobserver and strain variability

Interobserver variability for STE measurements are shown in Table 2. Smoothing had a very limited effect on the results, although agreement for most measurements was somewhat better with maximum compared to minimum smoothing. Poorest agreement was found for posterior wall strain with minimum spatial and temporal smoothing. In all three directions, independent of the degree of smoothing, agreement was in general very good for strain in the anterior segment as well as for global strain.

**Table 2:** Effect of smoothing parameters on interobserver variability for STE strain measurements in the radial, circumferential and longitudinal direction

	**MINIMUM**		**DEFAULT**		**MAXIMUM**	
	**Mean difference**	**R**_**ic**_	**Mean difference**	**R**_**ic**_	**Mean difference**	**R**_**ic**_
Radial direction						
Anterior segment	-2.2 ± 5.7	0.98	-1.9 ± 5.3	0.98	-1.6 ± 5.7	0.98
Posterior segment	4.4 ± 7.1	0.61	3.3 ± 6.3	0.68	2.0 ± 5.2	0.75
Global	-0.3 ± 3.9	0.97	0.1 ± 4.1	0.97	0.1 ± 4.0	0.97
Circumferential direction						
Anterior segment	0.4 ± 2.4	0.95	0.1 ± 4.1	0.97	0.1 ± 4.0	0.97
Posterior segment	0.3 ± 3.3	0.74	-0.8 ± 2.6	0.73	-0.9 ± 2.1	0.78
Global	-0.6 ± 1.4	0.97	-0.8 ± 1.5	0.96	-0.8 ± 1.1	0.97
Longitudinal direction						
Anterior segment	0.5 ± 3.9	0.85	-0.4 ± 1.7	0.97	-0.7 ± 1.7	0.97
Posterior segment	1.9 ± 3.5	0.82	1.3 ± 2.4	0.85	1.1 ± 2.4	0.85
Global	0.2 ± 2.4	0.88	0.3 ± 2.4	0.87	0.1 ± 2.1	0.90

Mean difference ± SD. n = 14. R_ic_ = intra-class correlation coefficient; Minimum = minimum spatial and temporal smoothing; Default = factory default spatial and temporal smoothing; Maximum = maximum spatial and temporal smoothing.

For all strain parameters, equal variance between the nine smoothing settings could be assumed (P > 0.05 by Levene’s tests). Furthermore, strain variance did not exceed interobserver variance for any of the measured strain parameters.

## Discussion

The present study investigated how different user controlled adjustments of spatial and temporal smoothing affected global and regional STE strain values. Consistent with our hypothesis, user dependent smoothing had a limited effect on the resulting strain estimates. The main findings were; (1) global and regional longitudinal strain was unaffected by user controlled spatial and temporal smoothing in both normal and ischemic myocardium and, (2) although variable effects of smoothing were found in the radial and circumferential direction, the different combinations of smoothing did for most situations only result in small changes in the strain values.

The current STE algorithm incorporates a preset amount of smoothing during analysis that is not controllable by the user [6,7]. However, when data processing is complete, the user is able to perform adjustments in the degree of both spatial and temporal smoothing. This study shows that the user controllable amount of smoothing had limited effect on the values in all three directions of strain estimation. The findings, however, do agree well with the fact that spatial and temporal smoothing levels in the short-axis view exceed those in the long-axis view for this software.

In the longitudinal direction, there is less out-of-plane movement of speckles, speckles are tracked over a longer distance and more knots are present in each segment compared to measurements in short-axis (Figure 1). These factors will increase the accuracy of the strain measurements. Thus, the true movement of speckles can be tracked more precisely in the longitudinal direction and could explain why the current strain estimates were not affected by user controlled smoothing. For regional measurements, however, a tendency for higher strain variability was observed with minimum spatial smoothing (Figure 7). This is also reflected by the higher interobserver variability for this setting (Table 2).

In short-axis, radial strain was more affected by smoothing than circumferential strain. Again, this could be explained by a reduced accuracy for strain estimation in this direction as speckle movements are measured over a shorter distance in the radial (limited by the wall thickness) compared to the circumferential direction (Figure 1A). This is in agreement with previous studies showing higher variability for radial strain measurements [20,21]. It also corresponds well with the general acceptance that feasibility of STE analysis is better for longitudinal and circumferential strain while it is more challenging for radial strain [4]. Additionally, there is a larger strain gradient across the wall in the circumferential and radial direction compared with the longitudinal direction [22-25]. This could in part also explain why smoothing did not alter longitudinal strain values while corresponding strain values in the circumferential and radial direction were affected.

In normal myocardium, global radial and circumferential strain decreased with increasing temporal smoothing (Figure 4A and D). Increasing temporal smoothing may decrease random noise, but also reduces the effective frame rate and thus the peak strain value. This is in accordance with a previous study on the effect of temporal smoothing on 2D peak velocity Tissue Doppler Imaging estimates [26], and could explain why all radial strain values decreased or tended to decrease with increasing temporal smoothing (Figures 4 and 5). Although minimum temporal smoothing is approximately equal to the time from one frame to the next, it is not possible to give specific frame rates for higher degrees of smoothing as this is based on an experimental constant not provided by the manufacturer.

Spatial smoothing did not have an effect on global strain in normal myocardium. This, however, was to be expected as measurements were already averaged over all segments. For regional strain, larger differences between anterior and posterior wall strain measurements were found in the radial direction (Figure 5A and B) compared to the circumferential direction (Figure 6A and B) in normal myocardium. This could explain why spatial smoothing affected strain values more in the radial than in the circumferential direction.

In general, the regional short-axis strain values seemed to be more affected by smoothing in the posterior than in the anterior wall (Figures 5 and 6). This is probably due to the lower beam density at the depth of the posterior wall (due to the sector format), thus resulting in more uncertainty in these speckle tracking strain estimates. Furthermore, out-of-plane motion of the speckles will also affect strain estimates more in the contracting posterior wall versus the non-contracting anterior wall during ischemia. This is reflected by the higher interobserver variability for the posterior wall segment (Table 2).

The current STE algorithm assumes a uniform thickness of the myocardium at end-systole when the ROI width is set [7]. Due to the large difference in regional wall thickening during ischemia, the anterior wall thickness was used to set the ROI width for this situation. With a thin anterior wall thickness, tracking of the posterior segment was performed so that the middle part of the posterior wall was included in the ROI. The observed changes in global and especially regional strain in the radial and circumferential direction during ischemia could therefore also in part be explained by a suboptimal ROI width for the posterior wall. One such effect could be the observation that increased spatial smoothing during ischemia resulted in a modest increase (more negative) in circumferential strain of the posterior wall (Figure 6D). This could possibly be explained by including more of the subendocardial part of the wall where the circumferential strain is highest [22-25].

Overall, the different combinations of STE smoothing settings did only result in small changes in the strain values and differences between most of the individual measurements remained within the variability of each strain estimate. Moreover, independent of the degree of smoothing, all strain values corresponded well to findings from previous studies in both normal and ischemic myocardium [10,18,20,27-30]. Different smoothing settings are therefore not likely to be a major contributor to the variances in strain reported in the literature [4]. Also, different smoothing settings did not affect strain variance. For all strain parameters, variance did not exceed the corresponding interobserver variance.

### Clinical implications

Different combinations of adjustable STE smoothing settings did for most cases only result in small changes in the strain values. In the radial direction, however, the differences between maximum and minimum strain values were larger. This is related to the larger transmural strain gradient in the radial direction and emphasizes the importance of proper spatial resolution and sampling for these measures. The current observations need to be examined further in a clinical feasibility study and for other interventions. STE algorithms from other vendors may respond differently to adjustments in spatial and temporal smoothing.

### Limitations

A methodological challenge associated with STE is the dependency of good image quality. These challenges were minimized in this open chest study. Acquisition was carried out with 8–10 MHz, resulting in B-mode resolution being better than with an adult cardiac probe (typically 3–5 MHz). Recordings were repeatedly of high quality and resolution, and all segments for STE analysis were accepted by the STE algorithm. This is also reflected by the low interobserver variability for most strain measurements in this study. Experimental studies allow a much higher degree of repeatability and standardization than what is possible in a clinical study. Changes in strain due to user controlled spatial and temporal smoothing may therefore be more prominent in a clinical setting, this question still remains open.

The purpose of the current study was to investigate the effect of different user controllable smoothing settings on the strain values. As such, the accuracy of the strain measurements themselves was not the main focus of the current study. Comparison of strain values against a gold standard method like sonomicrometry was therefore not performed. However, the current STE algorithm has been shown to perform accurately when compared to sonomicrometry under similar conditions [31].

Epicardial placement of the ultrasound probe could affect cardiac function and strain measurements. However, hearts were monitored by a large number of hemodynamic variables including HR, LVP and CI. If changes in these parameters occurred during echocardiographic recordings, the probe was temporarily removed and hearts were left undisturbed until normalization of these parameters. We are therefore confident that no major changes in hemodynamics occurred during our recordings. We thus believe this effect was small.

In normal myocardium, higher radial strain was found in the anterior compared to the posterior wall (Figures 3 and 5). This was also the case for wall thickening. These findings could be due to methodological aspects, as discussed in more detail elsewhere [18]. However, highest anterior wall thickening/radial strain could also in part be a physiological normal finding as MRI studies have demonstrated similar findings in the normal human left ventricle [32,33].

This study investigates one commonly used STE algorithm. Results are not validated for other available STE algorithms. However, the current results should be relevant for other STE algorithms using cubic spline smoothing for smoothing purposes.

This study included only seven animals. However, significant changes in several of the strain values were found, indicating a sufficient number of observations. Increasing the study population could have resulted in more significant changes as well as more robust estimates. The overall finding of this study, however, that smoothing parameters only resulted in small changes in most of the strain values, would not change.

## Conclusion

Global and regional longitudinal strain was unaffected by adjustments of user controlled spatial and temporal smoothing in both normal and ischemic myocardium when analyzing STE strain with the current software. Furthermore, variable effects of smoothing were found for strain parameters in the radial and circumferential direction. The different degrees of spatial and temporal smoothing did not affect strain variance. For all strain parameters, variance remained within the corresponding interobserver variance.

## Competing interests

All authors declare that they have no competing interests concerning this study.

## Authors’ contributions

All authors participated in the initiation and design of the study. All authors except JJH participated in the experiments and data collection. All authors participated in data analysis and interpretation of the results. All authors read and approved the final manuscript.
